# Supplementary material for: Auto-CORPus: A Natural Language Processing Tool for Standardizing and Reusing Biomedical Literature
Source: Front Digit Health. 2022 Feb 15;4:788124. doi: 10.3389/fdgth.2022.788124 (PMC8885717; doi:10.3389/fdgth.2022.788124)
Supplement: Supplementary file 1 [file Data_Sheet_1.PDF]

1 Supplementary Materials

There exists no equivalent to the GWAS Central database for MWAS/metabolomics studies, therefore a best effort was made to create a collection of publications that span multiple publishers, journals and focus on a variety of disease traits.

1.1 PubMed Central (PMC) search terms

We searched PMC in March 2020 for OA publications using the following search terms to appear in the title or abstract for the field (metabolomics, metabolome, metabolomic, metabolome-wide, metabonomics, metabonome, metabonomic, metabonome-wide, lipidomics, lipidome, lipidomic, lipidome-wide, metabolites, metabolite, metabolic profiling, metabolic phenotyping), type of samples (urine, urinary, blood, serum, plasma, faecal, faeces, cerebrospinal fluid, CSF, biofluid, stool, feces, fecal), type of technology (nmr spectroscopy, nuclear magnetic resonance, NMR, mass spectrometry, LCMS, GCMS, UPLCMS, LC-MS, GC-MS, UPLC-MS, CEMS, CE-MS), human studies (human, patients, subjects, participants) – all using an ‘AND’ statement – and exclude terms (using a ‘NOT’ statement) outside the scope (mouse, mice, rat, rats, dog, dogs, animal, cell culture, dose, review, proteomics, diet, proteomic, proteome, transcriptomics, transcriptomic, transcriptome).

These search terms were combined with either search terms targeting specific journals that commonly publish MWAS/metabolomics studies (Analytical Chemistry, Journal of Proteome Research, Analytica Chimica Acta, Journal of Chromatography A, Metabolites, Scientific Reports, PLOS One, Analytical and Bioanalytical Chemistry, Metabolomics, Proceedings of the National Academy of Sciences of the United States of America) or with specific disease trait terms in the title/abstract. For example, for cancer the additional search terms consist of: cancer, tumor, tumour, cancerous, carcinogen, carcinogenic, carcinoma, leukaemia, leukemia, leukaemic, leukemic, lymphoma, malignancy, premalignancy, pre-malignancy, melanoma, metastasis, sarcoma, adjuvant, neoadjuvant, chemotherapy, chemo therapy, chemo-therapy, malignant, premalignant, pre-malignant, precancerous, pre-cancerous, adenocarcinoma, metastatic. The same approach was used for publications relating to gastrointestinal diseases, metabolic syndrome, sepsis and, neurodegenerative, psychiatric, and brain illnesses.

The full-text OA publications were downloaded in HTML format after duplicates (e.g. a ‘cancer’ study from one of the targeted journals) were removed. This resulted in a total of 1,241 unique publications that are included in the OA dataset.

2 Supplementary Methods

2.1 Image processing

Our current work includes the development of a pipeline of image processing operations to convert table images in JPG or PNG formats to table JSON. The three stages of the pipeline are cell detection, text recognition and table structure analysis.

2.1.1 Cell detection

The OpenCV library (https://github.com/opencv/opencv/tree/4.5.3) is used to preprocess images prior to cell detection. Image binarization is used to remove background colours, font colours and provide a high contrast between the target text and the background. Line detection is used to remove all grid lines from the table image, with the OpenCV minimum line length parameter configured to distinguish between table grid lines and short line characters such as “i”, capitalized “I” or “l”. Mathematical morphology processing is then used to identify cell text by conducting quantitative description and analysis of geometric shapes and structures based on set theory. Text blocks of interest are processed by thickening the black texts and connecting the discontinuous parts close to each other to blur black pixel regions, creating black “smudges” that identify the location of each text block. Rectangular boxes are drawn around each block to create individual cells that overlap with the original table cells and their spatial positions are indexed by the rectangle boundary’s horizontal and vertical coordinates. Supplementary Figure 3 illustrates the cell detection steps.

2.1.2 Text recognition

Google’s Tesseract optical character recognition (OCR) engine is used through the Python-tesseract wrapper (https://pypi.org/project/pytesseract/) to recognise and extract text from the preprocessed table images. Tesseract is based on Long Short-Term Memory (LSTM) neural networks which enables users to train models aligned to their use cases. PyTesseract provides more than 100 trained libraries in different languages and we found that using the PyTesseract English language “eng.traineddata” library, the OCR engine correctly recognised 53.14% of cell text from the images in the publisher dataset (33,273 cells). Cells containing special characters such as superscripts, subscripts and Greek characters were rarely recognised. We trained a dataset using biomedical data to fine-tune the PyTesseract model, resulting in the OCR engine correctly recognising 87.92% (median, interquartile range: 86.14-90.58%) of cell text from the table images in the publisher dataset. Superscript and subscript characters were present in most cells not recognised.

2.1.3 Table structure analysis

The text from each cell is analysed to determine the cell function and generate the structured table JSON output. The algorithm reads all of the cells line by line, from top-left to bottom-right, and creates a map of their relative positions. Spatial positioning and regular expression rules are then used to identify the table identifier, title, caption, footer and column headers. Super rows and index columns are identified and recognised as table section divisions (the end of a previous section and start of new section) and the cell text is included as the section title in the table JSON.

2.2 NER on GWAS publications

To demonstrate the potential applicability of Auto-CORPus for text mining of the biomedical literature we used the GWAS publications from the OA dataset. This subset contains 1,200

publications that are present in the GWAS Central database (https://www.gwascentral.org/) which allows cross-referencing with manually extracted entities. Assigned randomly, 700 publications are used as a training set and 500 as a test set.

Prior work on GWAS publications from ourselves (unpublished), and from others (1), has shown phenotypes, *P*-values and single nucleotide polymorphisms (SNPs) can be extracted using regular expression matching. Here we focus on 5 different branches of important information that can be extracted about each study: platform recognition (company names), total number of SNPs assayed (total SNPs), sequencing technology used (exact array/assays), how quality control (QC) was performed (presence of QC, software version number), and whether imputation was performed (imputation including possible negation). The training set was created using data from GWAS Central which was manually curated (platform (list of strings), total SNPs passing QC (numbers in different formats) and imputation (binary)) and used for annotation, as well as a manually created list of entities for each of the categories. The sequencing technology (exact assay) was annotated by combining a search for the platform name, specific words (array, chip, etc.) with a regular expression algorithm to look for combinations of letters and numbers in sequence. For each of these branches, sentences containing relevant entities for training were extracted from the Auto-CORPus BioC JSON output to create a training set for each branch, for this we restricted ourselves to the (statistical) methods/materials sections only (IAO:0000317, IAO:0000633, and IAO:0000644).

SpaCy 3.0 (https://spacy.io/models/en#en_core_web_lg) was used to develop each of the algorithms using word embedding and a multilayer convolution neural network (CNN) along with residual connections (2) and models were optimized for accuracy (over speed). The maximum batch size was set to 1000 and the proprietary spaCy quickstart configuration file was deployed for every single branch model, allowing for unlimited epochs until the model scoring plateaus.

3 Supplementary Results and Discussion

The NER tools developed for the five branches showed promising performance for automatically extracting GWAS entities from biomedical literature with F1-scores 0.82-1.00 (Supplementary Table 2). Supplementary Figure 4 shows examples of the pipeline output (after merging output for each branch) for different sentences from the test set. The branches with the best performance were the platform, quality control and imputation entities (all F1-scores over 0.95). The least successful was the algorithm to recognise the exact assays used with an F1-score of 0.82. This is likely due to the wide variety of sequencing arrays available and the constant development over time, including in the naming conventions.

4 Supplementary References

1. Kuleshov V, Ding J, Vo C, Hancock B, Ratner A, Li Y, et al. A machine-compiled database of genome-wide association studies. *Nat Commun* (2019) **10**:3341-x doi: 10.1038/s41467-019-11026-x [doi].

2. Digan W, Névéol A, Neuraz A, Wack M, Baudoin D, Burgun A, et al. Can reproducibility be improved in clinical natural language processing? A study of 7 clinical NLP suites. *J Am Med Inform Assoc* (2021) **28**:504-15 doi: 10.1093/jamia/ocaa261 [doi].

5 Supplementary Figures

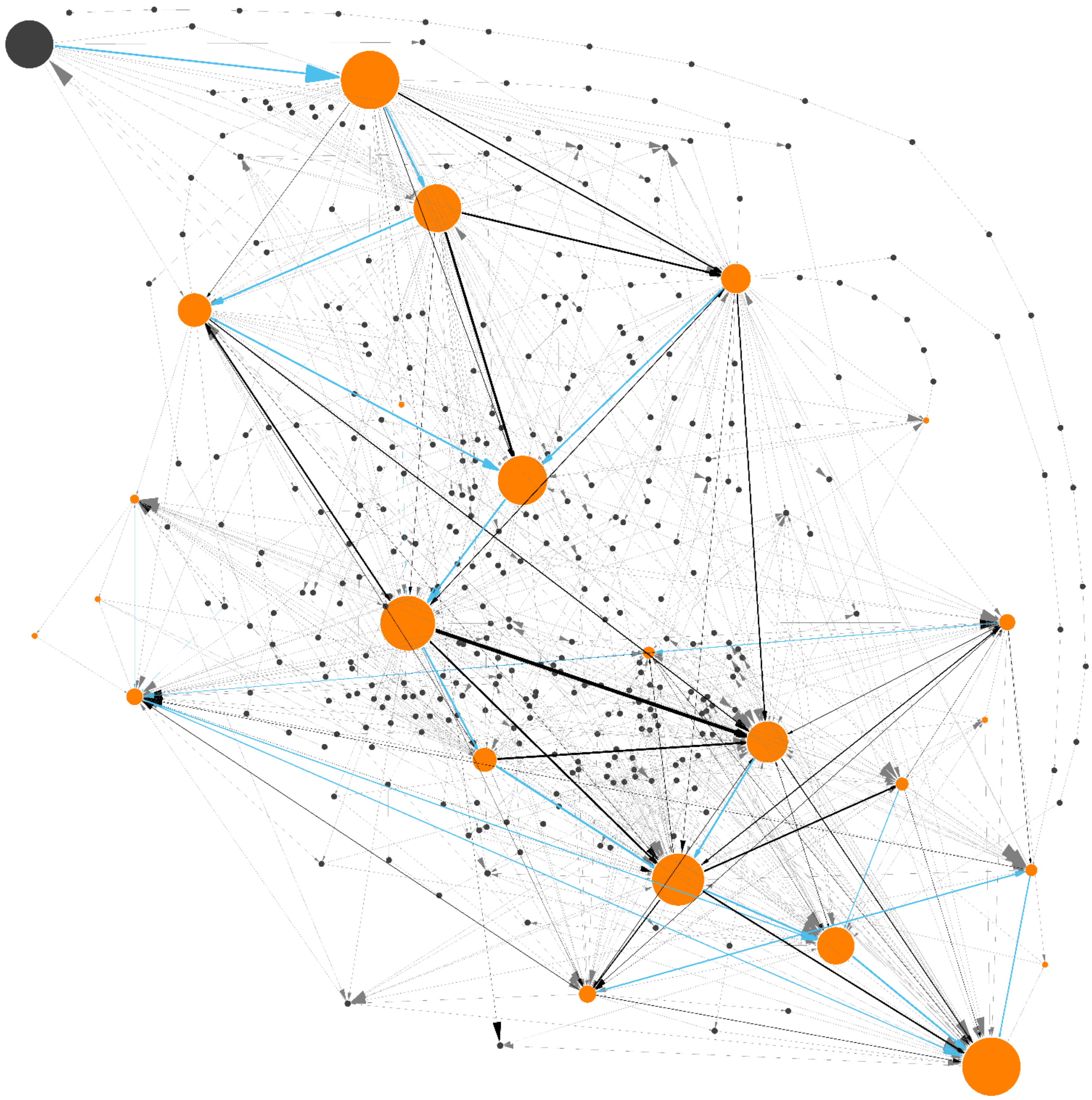

Supplementary Figure 1. Digraph generated from analyzing section headers from 2,441 Open Access publications from PubMed Central. The digraph of the v2020-06-10 IAO model consists of 372 unique nodes, of which 24 could be directly mapped to section terms (in orange) and the remainder are unmapped headers (in grey), and 806 directed edges. Relative node sizes and edge widths are directly proportional to the number of publications with these (subsequent) headers. Blue edges indicate the edge with the highest weight from the source node, edges that exist in fewer than 1% of publications are shown in light grey and the remainder in black.

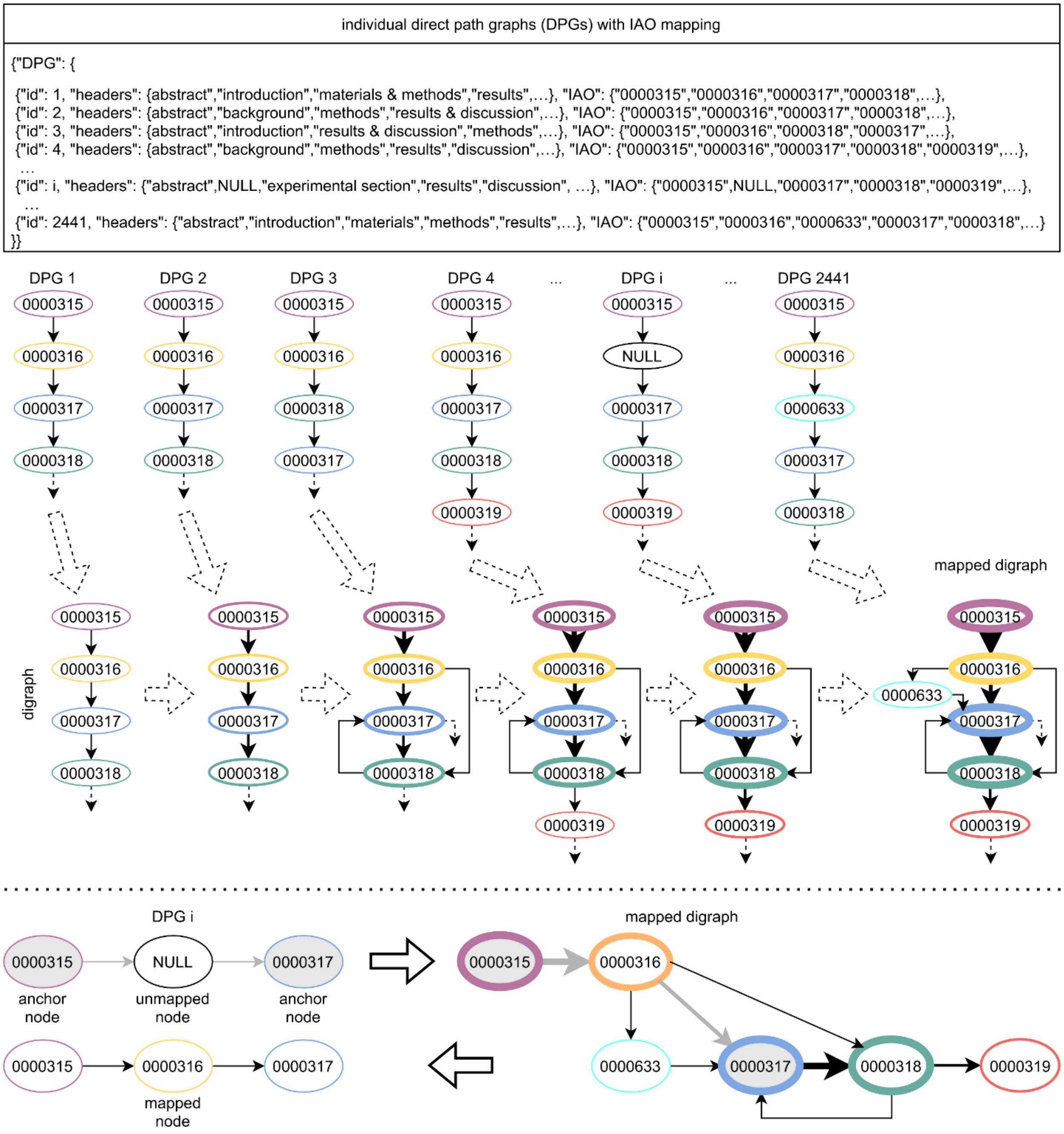

Supplementary Figure 2. Pictographic example of combining directed path graphs (DPGs) into a digraph and using anchor nodes to map unmapped headers. Top panel illustrates the data structure of the individual DPGs extracted from the OA dataset including the header and Information Artefact Ontology (IAO) term they are mapped to. The middle panel illustrates how DPGs are combined into the digraph. The bottom panel indicates how anchor headers (nodes) are used to predict the IAO term of unmapped nodes based on the mapped digraph (see Figure 3 and Supplementary Figure 1) to find the shortest path between the mapped nodes and assigning unmapped nodes to nodes in the graph. Paths in the digraph between anchor nodes that are shorter than the DPG are mapped to the first anchor node.

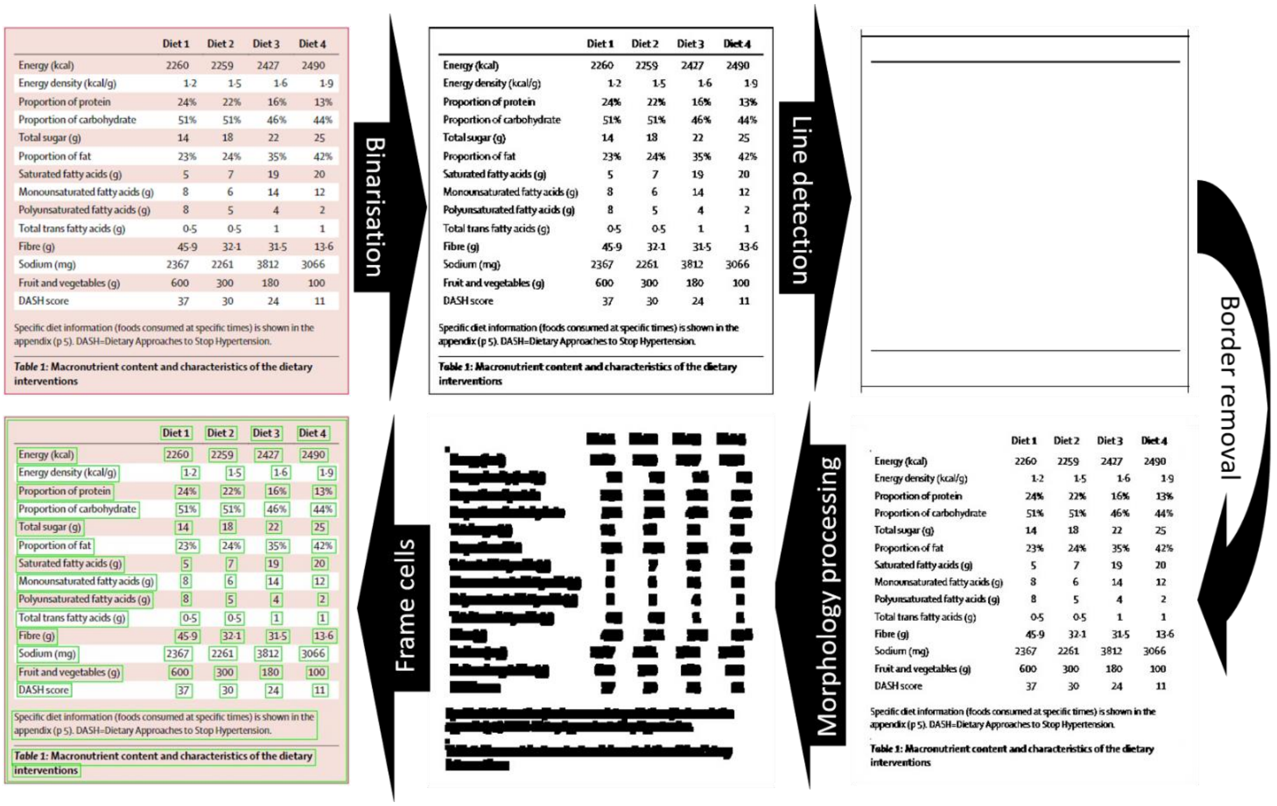

Supplementary Figure 3. The image processing steps involved in cell detection. Binarization removes colour shading from table images to represent tables with black and white pixels only. Line detection is followed by the removal of horizontal and vertical grid lines in the image to reduce noise that could affect the morphology process. Text blocks are represented as blackened “smudges” to aid recognition, and finally, all cells containing text are identified and framed. The table is reproduced here under a Creative Common license (doi: 10.1016/S2213-8587(16)30419-3).

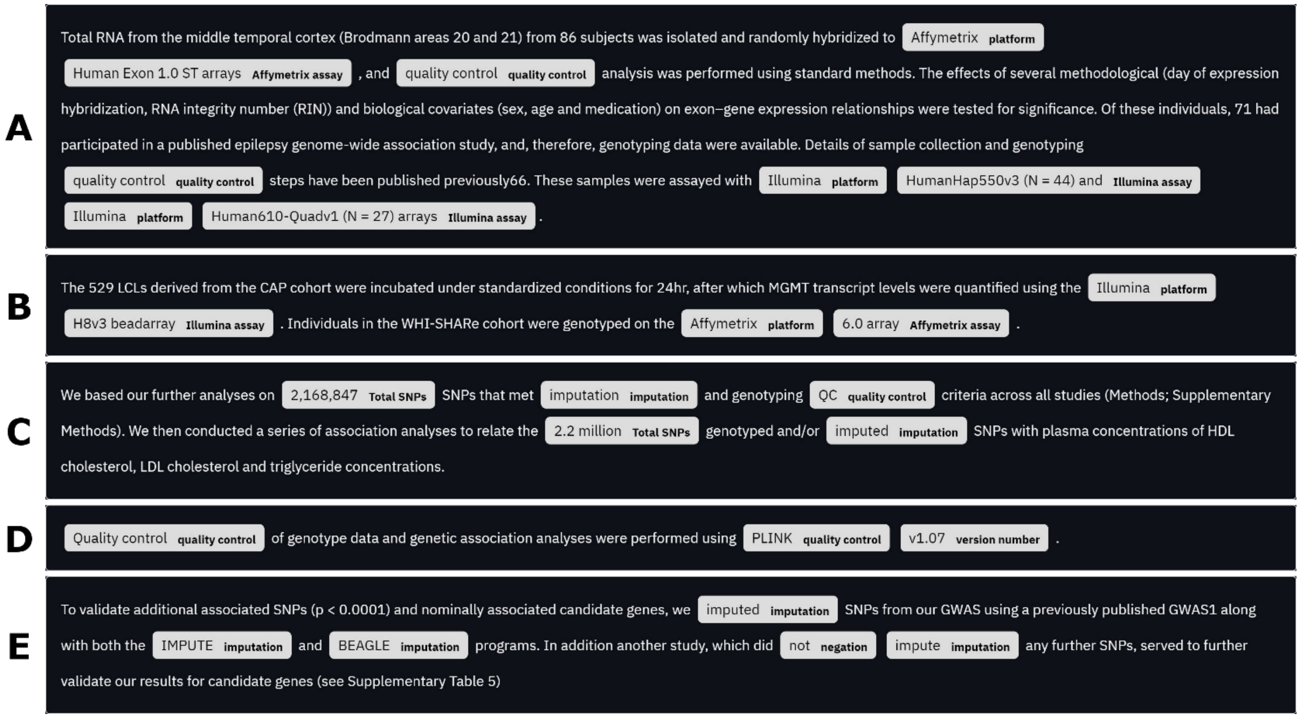

Supplementary Figure 4. Example sentences from the GWAS publications test set (n=500) with recognized entities tagged. These examples combine the output from each branch for the individual NER tasks. (A) Details recognised platforms (platform), sequencing technology used (Affymetrix assay, Illumina assay), and presence of quality control (quality control). The latter 4 entities recognized were found as a ‘single’ Illumina assay but split after post-processing and combining with the platform NER algorithm output. (B) Details recognised platforms (platform) and sequencing technology used (Affymetrix assay, Illumina assay). (C) Details total number of SNPs (Total SNPs), whether imputation was used (imputation), and presence of quality control (quality control). (D) Details presence of quality control (quality control, version number). (E) Details whether imputation was used (imputation, negation). Note: software programs for quality control and imputation were labelled as the main category (quality control, imputation).

6 Supplementary Tables

Category	Journal(s)	Observations
Mapping of unrecognised headers to IAO terms	Frontiers in Physics<br><br>Frontiers in Psychology<br><br>Physical Review Letters<br><br>Psychological Bulletin<br><br>Psychological Science	All paragraphs (with headers: ‘Comorbidity’, ‘Existing Theoretical Models’, ‘A Multiple Pathways Model’, ‘Descriptive and Developmental Variables’, ‘Assessment’, and ‘Treatment Response’) between ‘Abstract’ and ‘Conclusions and Future Directions’ mapped to introduction section, materials section, results section, and discussion section (PMC4006306)<br><br>‘Gender Differences in Depression across Nations’, ‘Additional Factors Influencing the Gender Difference in Depression’ and ‘The Current Study’ were correctly mapped to introduction section (PMC5532074)<br><br>‘ML Models’ was correctly mapped to methods section (PMC6941671)<br><br>‘Relevant models of time-dependent diffusion’ was correctly mapped to introduction section (PMC6296484)<br><br>‘METHODS AND MATERIALS’ was correctly mapped to both methods section and materials section (PMC6820857) [a]<br><br>All paragraphs (with headers: ‘Study 1’ and ‘Study 2’) between ‘Abstract’ and ‘Discussion’ mapped to introduction section, materials section, and results section (PMC7366427)<br><br>‘Transparency’ mapped to footnotes section (PMC7366427, PMC7370246, PMC7883001) [b]<br><br>‘Accelerated Development/Biological Aging’ and ‘The Current Study’ were correctly mapped to introduction section (PMC7484378)<br><br>All paragraphs (with headers: ‘Model.—’, ‘Strong HSF.—’, ‘Subsector thermalization and integrability.—’, and ‘HSF in gauge theory.—’) between ‘Introduction.—’ and ‘Conclusion.—’ mapped to materials section, results section, and discussion section (PMC7693131)<br><br>Between introduction (no header but mapped) and ‘Discussion’: ‘Experiments 1 to 4’ mapped to introduction section, ‘Experiments 5 and 6’ mapped to methods section (PMC7883001)<br><br>‘Publisher’s Note’ (PMC8566543, PMC8595922) was correctly mapped to notes section<br><br>All paragraphs (with headers: ‘College Students' Entrepreneurial Psychology’ and ‘Experimental Analysis of Wavelet NN’) between ‘Introduction’ and ‘Conclusions’ mapped to materials section, results section, and discussion section (PMC8595922)
Mapping of paragraphs in articles without headers	Nature Physics<br><br>Physical Review Letters	All paragraphs between ‘Abstract’ and ‘Conclusions and implications’ mapped to introduction section, materials section, results section, and discussion section, these were not mapped to the methods section as one appears at the end and was separately mapped (PMC5152624)<br><br>All paragraphs between ‘Abstract’ and ‘Acknowledgements’ mapped to introduction section, materials section, results section, discussion section, and supplementary material section (PMC5525544, PMC6649682, PMC7489308)<br><br>All paragraphs mapped to introduction section due to methods section appearing at the end as only section with header (PMC6071846, PMC7116451)
Mapping of non-sequential headers to same IAO term	Psychological Science	‘Supplementary Material’ and ‘Appendix’ were both correctly mapped to supplementary material section while appearing ‘Acknowledgments’ appears in between these headers in the directed path graph (PMC7370246)

Supplementary Table 1. Mapping of headers from non-GWAS/MWAS publications from 3 physics and 3 psychological journals to IAO terms. Key: [a] This synonym was not identified in the GWAS/MWAS publications; [b] This term may be a candidate for a separate document category.

Branch (entity tags)	F1-score
Platform technology (platform)	1.00
Total number of SNPs (Total SNPs)	0.89
Assay name (Illumina assay, Affymetrix assay, Perlegen assay)	0.82
QC (quality control, negation, version number)	0.98
Imputation (imputation, negation, no imputation) [a]	0.95

Supplementary Table 2. F1-score for each of the 5 branches of GWAS NER evaluated on 500 publications from the test set. [a] The 'no imputation' tag is distinct from negation and focuses on words such as 'unimputed' and 'non-imputed', whereas negation focuses on entities such as 'did *not* perform' and 'was *not* used' that suggests ambiguity (i.e. relationship to concepts needs to be inferred). These tags are given as output of our pipeline (also see Supplementary Figure 4).
